# Author Correction: Fingerprinting the Cretaceous-Paleogene boundary impact with Zn isotopes

**DOI:** 10.1038/s41467-021-26377-7

**Published:** 2021-10-11

**Authors:** Ryan Mathur, Brandon Mahan, Marissa Spencer, Linda Godfrey, Neil Landman, Matthew Garb, D. Graham Pearson, Sheng-Ao Liu, Francisca E. Oboh-Ikuenobe

**Affiliations:** 1 Geology Department, Juniata College, Huntingdon, PA USA; 2 Earth and Environmental Science, James Cook University, Townsville, QLD Australia; 3 Department of Geosciences and Geological and Petroleum Engineering, Missouri University of Science and Technology, Columbia, MO USA; 4 Department of Earth and Planetary Science, Rutgers University, New Brunswick, NJ USA; 5 Division of Paleontology, American Museum of Natural History, New York, NY USA; 6 Department of Earth and Environmental Science, City University of New York, Brooklyn College, Brooklyn, NY USA; 7 Department of Earth and Atmospheric Sciences, University of Alberta, Edmonton, AB Canada; 8 China University of Geosciences, Beijing, China

**Keywords:** Geochemistry, Geochemistry

Correction to: *Nature Communications* 10.1038/s41467-021-24419-8, published online 05 July 2021.

The original version of this Article contained an error in the ‘Methods’, which incorrectly read ‘The error of the standard compared to itself throughout the four sessions is ±0.05‰ and is considered a conservative estimation of error as duplicate analyses of the same solutions and complete procedural duplicates of the USGS BVHO-2 rock standard (+0.01 ±0.03‰, 2σ, *n* = 8, overlapping values found in) are significantly less.’ The correct version states ‘(+0.28 ±0.03‰, 2σ, *n* = 8, overlapping values found in)’ in place of ‘(+0.01 ±0.03‰, 2σ, *n* = 8, overlapping values found in)’.

This has been corrected in both the PDF and HTML versions of the Article.

