# Supplementary material for: Fingerprinting the Cretaceous-Paleogene boundary impact with Zn isotopes
Source: Nat Commun. 2021 Jul 5;12:4128. doi: 10.1038/s41467-021-24419-8 (PMC8257607; doi:10.1038/s41467-021-24419-8)
Supplement: Supplementary file 1 — Supplementary Information [file 41467_2021_24419_MOESM1_ESM.pdf]

Supplementary Information for

Fingerprinting the Cretaceous-Paleogene boundary impact with Zn isotopes

Ryan Mathur[1*], Brandon Mahan[2], Marissa Spencer[3], Linda Godfrey[4], Neil Landman[5], Matthew Garb[6], Sheng-Ao Liu, Francisca E. Oboh-Ikuenobe[3]

1. Juniata College, Geology Department, PA, USA
2. James Cook University, Earth and Environmental Science, Townsville, AUS
3. Missouri University Science and Technology, Geosciences, MO USA
4. Rutgers University, Earth and Planetary Science, NJ USA
5. American Museum of Natural History, Paleontology, NY USA
6. CUNY, Brooklyn, Geosciences, NY USA
7. China University of Geosciences, Beijing, China

*Ryan Mathur
Email: mathurr@juniata.edu

This file includes:

Figures Supplementary Figure 1

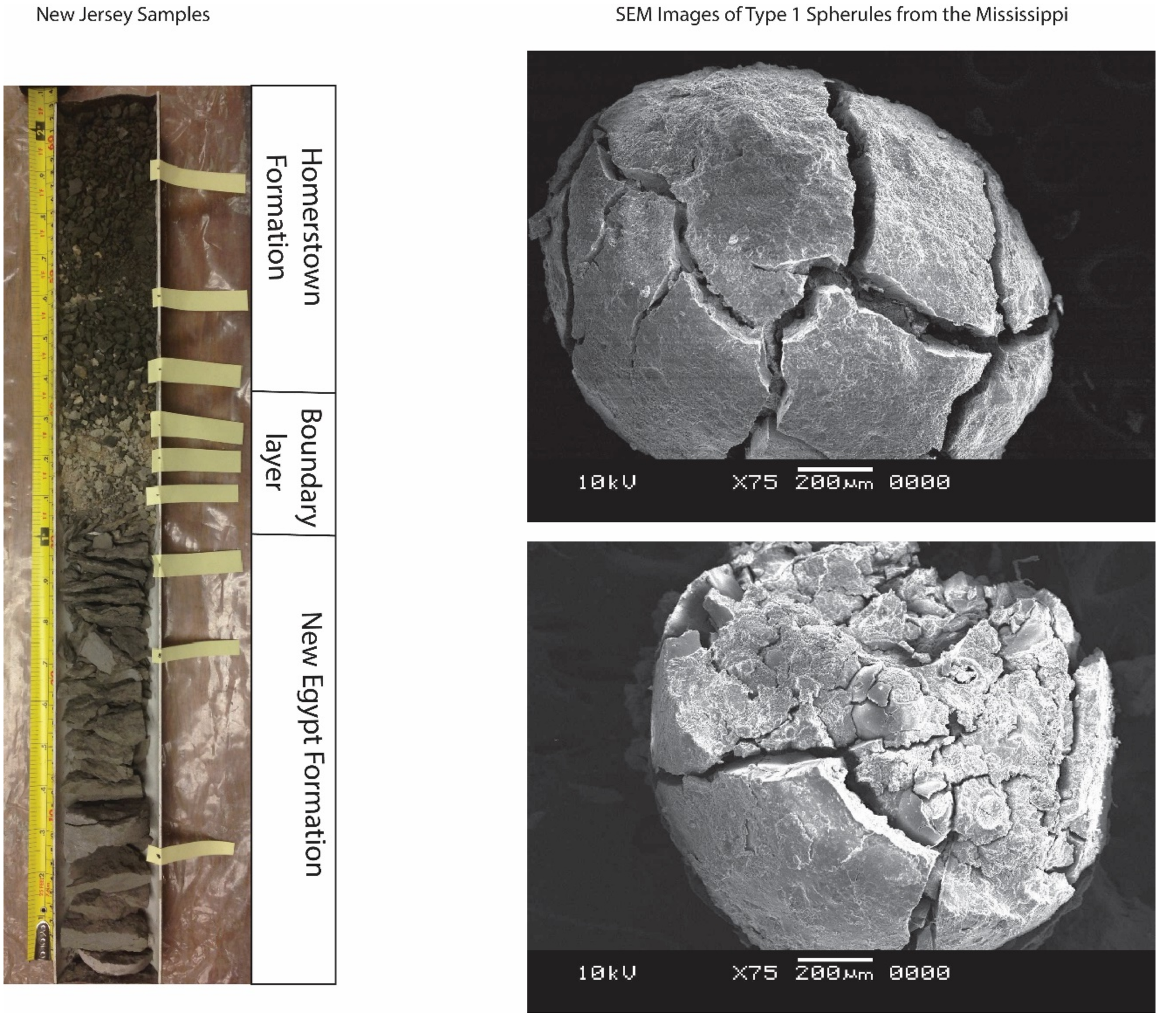

Fig. Supplementary Figure 1. These images provide examples of materials analyzed for the study. Figure A) displays the samples from the New Jersey Drill core with yellow tabs indicating sample position and B) provides SEM images of the Type 1 spherules found in the boundary layer from Mississippi.
